# Components of Unrealistic Optimism of College Students: The Case of the COVID-19 Pandemic

**DOI:** 10.3389/fpsyg.2021.763581

**Published:** 2021-12-13

**Authors:** Yohanan Eshel, Shaul Kimhi, Hadas Marciano, Bruria Adini

**Affiliations:** ^1^Department of Psychology, University of Haifa, Haifa, Israel; ^2^Stress and Resilience Research Center, Tel-Hai College, Tel-Hai, Israel; ^3^The Institute of Information Processing and Decision Making (IIPDM), The Ergonomics and Human Factors Unit, University of Haifa, Haifa, Israel; ^4^Department of Emergency and Disaster Management, School of Public Health, Sackler Faculty of Medicine, Tel Aviv University, Tel Aviv, Israel

**Keywords:** COVID-19, perceived risks, unrealistic optimism, distress symptoms, individual resilience, well-being, college students

## Abstract

College students are among the most strongly affected populations by the coronavirus disease-2019 (COVID-19) pandemic because of uncertainty regarding academic success, future careers, and social life during their study period. Their mental health and behavior may dramatically be impacted. The study examined an unrealistic optimism of Israeli college students in assessing the health, security, and economic risks during the pandemic, and the contributions of these perceived risks to the prediction of psychological coping responses, such as well-being, and coping suppressing response of anxiety, expressed during this pandemic. Using social networks, a questionnaire was disseminated to students during the third lockdown that was implemented in Israel because of the pandemic. Depressive and anxiety symptoms, perceived threats, resilience, well-being, hope, and morale were measured using a structured quantitative questionnaire. First, we hypothesized that the three perceived risks would be inversely rated, so perceived health risk would be rated lowest, and perceived economic risk would be rated highest. The second and third hypotheses claimed that psychological coping responses articulated along this pandemic would be predicted by all these perceived risks, as well as the observance of pandemic precaution rules. The fourth hypothesis suggested that the three investigated perceived risks will positively and significantly correlate with each other. The results generally supported the hypotheses and indicated that the unrealistic optimism process was employed quite consistently by the participating students.

## Introduction

The coronavirus disease-2019 (COVID-19) pandemic has a dramatic impact on the mental health and behavior of people. Studies on COVID-19 risk perception and perceived cognitive and emotional dimensions found that it was associated with higher levels of frustration, confusion, inadequacy, uncertainty, anxiety, anger, and loneliness (Lanciano et al., 2020), as well as lower levels of coping, well-being, and finding meaning in life (Krok and Zarzycka, 2020). A comprehensive study on the antecedents of this risk perception, conducted in 10 countries around the globe (Dryhurst et al., 2020), found that this perception was significantly influenced by cognitive, emotional, social, and cultural factors, as well as direct and indirect experiences with the virus.

The study was conducted at the beginning of the year 2021, after a peak of the COVID-19 pandemic, during which an overall lockdown policy was directed, requiring the entire Israeli population, except for employees in vital services, to remain at home 24/7. The decrease of this pandemic was most likely achieved due to a national COVID-19 vaccination campaign (Leshem and Wilder-Smith, 2021). Vaccinations were widely available from December 2020, according to a prioritization schedule determined by the Israeli Ministry of Health (MOH). During the early phases of the distribution process, individuals considered as being at high risk for COVID-19 were prioritized for vaccination, such as those older than 60 years, nursing home residents, healthcare workers, and those with severe comorbidities. Later on, the vaccination campaign was gradually expanded until all individuals aged 16 years and older were eligible to receive the vaccine. The national vaccination campaign has led Israel to have one of the highest rates of vaccinated individuals per capita. As of February 24, 2021, 68.7 and 48% of the eligible population was vaccinated with one or two doses of vaccine, respectively (Rossman et al., 2021). This successful campaign did not prevent the immediate effects of the crisis on the Israeli economy, in the form of rising public expenditure and levels of unemployment (Bental and Shami, 2020a,b). In a breakdown by sector, expenditure in gas stations declined by about half during the first lockdown and by 21% during the second. Expenditure in the restaurant section was reduced to one-third its normal level, and in the hotel and leisure industries, expenditure was reduced to one-quarter and one-half its normal level, respectively. In grocery store chains, expenditure rose by more than one-third during the first lockdown. Additionally, many Israelis became unemployed or were put on unpaid leave because of the COVID-19 crisis. After declining between the lockdowns, the rate of unemployment insurance recipients rose again during the second lockdown and reached 240,000 additional recipients.

College students are among the most strongly affected populations by this pandemic because of additional uncertainties regarding academic success, future careers, and social life during their study period, among other concerns (Son et al., 2020). Furthermore, college students feel increased stress levels, anxiety, and depressive symptoms as a result of the uncertainty of university education, technological concerns of online courses, social isolation, decreased family income, and future employment. These impacts have been observed in universities across the world (Aristovnik et al., 2020). Among the most commonly reported effects were lack of motivation, anxiety, stress, and isolation, as well as social distancing (Browning et al., 2021). Efforts to reduce these worries and anxieties by psychological means often employ unrealistic optimism (Breznitz, 1983).

### Unrealistic Optimism

Research has found that under threatening and uncertain conditions, individuals tend to falsely reduce the perceived threat of adversities faced by them. This tendency is called “optimism bias” or “unrealistic optimism” (Shepperd et al., 2015). Such personal risk reduction relies on the belief concerning the likelihood that an adverse event will hurt the individual and the severity of that event (Floyd et al., 2000; Milne et al., 2000). Unrealistic optimism is defined as the “tendency for people to believe that they are less likely to experience negative events and more likely to experience positive events than are other people” (Shepperd et al., 2002, p. 65). Another mode of unrealistic optimism refers to an unjustified belief that a personal outcome, such as exam grades, will be more favorable than it should be according to some quantitative objective standards (Shepperd et al., 2017). This “better than average effect” of unrealistic optimism has been previously found in students’ belief that positive events are more likely to happen to them while negative events are less likely to happen to them, compared to the average student (Campbell et al., 2007). A cross-cultural research presents a more complicated phenomenon: unrealistic optimism as a form of self-enhancement is shaped by status (Sissons Joshi and Carter, 2013). The health belief model (HBM) investigates unrealistic optimism in relation to the risk of occurrence of selected health problems, and the extent to which it leads the failure of people to engage in positive behaviors that will promote their health and well-being (e.g., Peterson and de Avila, 1995). Studies on cancer threats, conducted on members of the general public, have, thus, found an optimistic bias pertaining to prostate cancer, for all HBM variables: risk and severity of prostate cancer and barriers to and benefits of screening (Clarke et al., 2000). An interplay of culture and socioeconomic circumstances was identified; for example, Indian participants have shown higher levels of optimism than English partakers in rating bad events. No such comparative unrealistic optimism has been found for English participants in good events and in the Indian sample it appeared only among members of higher socioeconomic conditions (Peterson and de Avila, 1995; Clarke et al., 2000). Similar unrealistic optimism has been found in a study on fraudulent transactions occurring *via* the Internet or automatic teller machines (ATMs). Results indicate that users have typically felt safe and secure while conducting financial transactions with the ATM, and that their behavior has reflected components of the HBM. They perceived the level of threat as low, mainly because they thought it unlikely that they would be victims of fraud and because of reduced sense of responsibility for any negative outcomes. Despite being aware of such fraudulent activities, they were not sure about the efficacy of behaviors designed to counteract fraud, and their potential efficacy (Davinson and Sillence, 2014).

Furthermore, security concerns among ATM users were not as high as concerns among Internet users, with Internet users appearing to take higher individual responsibility of their personal technologies in more private spaces. Thus, it was shown that unrealistic optimism can determine beliefs on health, romantic relationships, and professional success (Scheier and Carver, 2008).

It has been argued that these beliefs are, in many cases, genuinely unrealistic and irrational, since they are often based on information that is less than rational (Jefferson et al., 2017). However, it is also claimed that although unrealistic optimism includes systematic tendencies to form beliefs that are biased and often false, it involves significant benefits as well (Taylor and Brown, 1994): it increases well-being, contributes to mental and physical health, and supports productivity and motivation (Bortolotti and Antrobus, 2015).

Three major potential explanations were offered by Shepperd et al. (2015) for unrealistic optimism: first, impression management or self-enhancement goals; second, belief of people that they are unlikely to experience unfavorable outcomes; third, people judging their likelihood of experiencing an event on the basis of how well they match their stereotype of the people who experience the relevant event. The explanation of these authors for unrealistic optimism claims that people tend to transform a comparative judgment into a personal judgment, so their perception of a personal risk is sometimes based merely on their personal feelings.

Two major general explanations have been offered for the persistence of unrealistic optimism in the face of contrary information. The first emphasizes on attention processes. Sharot et al. (2011) claim that it perseveres through selective attention for new information that confirms positive beliefs and disregards information that contradicts these beliefs. Moreover, these beliefs are accepted as truth by the individual (Jefferson et al., 2017). The phenomenon of unrealistic optimism is widespread and is applied in many situations, ranging from health to perceptions of different risky situations (Reyes-Velázquez and Sealey-Potts, 2015). It appears that unrealistic optimism is so persistent because of its positive psychological contribution to the individual. Research has shown that such optimism can promote resilience and motivate adaptive responses to adversity (McKay, 2009; Johnson and Fowler, 2011; Kleiman et al., 2017). Thus, it was found that individuals who are unrealistically optimistic about their future success tend to cope with stressful conditions better (Colombo et al., 2020).

The second explanation for unrealistic optimism argues that it represents a partial denial of a dangerous situation, in which a life-threatening risk is rated lower than less risky threats (Kirscht et al., 1966; Solomon and Prager, 1992). An extensive review of this issue concludes that partial denial is a very common phenomenon in the case of illness (Livneh, 2009, II). New patients may deny their cardiac illness (Covino et al., 2011), and chronic patients, who are well aware of their physical condition, may be partly reluctant to acknowledge health-related information and its effect on their lives (Kortte and Wegener, 2004). The prevalence of lowering the perceived COVID-19 health risk seems to support the claim that this partial denial of threats is an emotional focused process, aimed at supporting individual adjustment to harmful and traumatic external events, which contributes toward supporting the resilience of Horowitz (1986). Breznitz (1983) and Lazarus (1983) have argued further that the advantages of partial denial are successful reduction of anxiety, stress, and other psychological symptoms, and raising life satisfaction and adjustment among most people who fear a serious illness.

We assume that despite the heightened public awareness of the potential negative health impacts of the COVID-19 pandemic, people will often use partial denial of its risks, in the form of unrealistic optimism, in order to reduce anxieties and foster goal persistence, positive affect, and hope (Shepperd et al., 2015). The prevalence of lowering the perceived COVID-19 health risk seems to support the claim that rather than being a pathological response, the partial denial of threats is an emotional focused process, aimed at supporting individual adjustment to harmful and traumatic external events, which contributes toward an adaptive behavior, supporting the resilience of people (Horowitz, 1986).

The effect of unrealistic optimism on the perceived risk of the COVID-19 pandemic was demonstrated, for instance, by a recent study in which an Italian and Swedish sample rated the pandemic risk lower than secondary risks associated with work and the institutional economy (Lanciano et al., 2020).

These findings raise an interesting question: are there realistic or rational components in unrealistic optimism, pertaining to the COVID-19 pandemic? By the same token, it should be expected that in ratings of three dangers, such as perceived risks of health, security, and economy, the deadliest perceived health risk will be rated lowest, and the perceived threatening but not lethal economic risk will be rated highest.

Previous research concluded that a lower rating of perceived health risk, as compared to lesser threats, is aimed at reducing the anxiety raised by a life-threatening adversity (Kirscht et al., 1966). Therefore, we assume that, facing a dangerous and uncertain condition that threatens their health, academic studies, and, perhaps, their professional future, students will probably attempt to reduce their level of anxiety by adopting the unrealistic optimism attitude that this pandemic is not as dangerous as it is presented by the media to the public.

An additional issue pertains to the predictors of psychological coping and coping suppressing responses expressed during the COVID-19 pandemic, such as well-being, resilience, and anxiety and/or depression. These responses were generally attributed mostly to the effects of the perceived health risk (e.g., Gautam et al., 2020).

### Predictors of Coping Responses

Research found that external circumstances and other risk sources that concern the general public are likely to impact adversity and pandemic risk perceptions (Ferrer and Klein, 2015). We claim, therefore, that the psychological coping responses expressed during the COVID-19 pandemic will be predicted concurrently by perceived health, security, and economic risks. These three perceived risks are supposed to positively and significantly correlate with each other.

We assume further that symptomatic psychological coping suppressing responses, such as anxiety, depression, and perceived academic stress, will be positively predicted by all the three perceived risks, since these responses are enhanced in anticipation of aversive events (Grupe and Nitschke, 2013). A different prediction pattern will characterize the positive psychological coping responses. A recent Israeli study (Gesser-Edelsburg et al., 2020) claimed that the two major perceived threats that currently concern the Israeli public are health and economic risks (Huynh, 2020). As the health risk perceptions increase, the evaluation of economic threat also tends to increase, and vice versa. Furthermore, the National Security Index of Israel shows that similar to the past few years, in 2020, the majority of the public believed that national security situation of Israel was fairly good (Israeli and Pines, 2021). In line with these findings, we assume that positive psychological coping responses like well-being and individual resilience will be negatively predicted by perceived health and economic risks, but not by perceived security risk. Because of the process of unrealistic optimism, the health risk will not be the major predictor of most of the psychological coping or coping suppressing responses expressed during the COVID-19 pandemic.

### Psychological Coping Responses

#### Distress Symptoms

The COVID-19 pandemic was negatively associated with psychological distress responses of grief, hopelessness, posttraumatic symptoms, panic attacks, stress, anxiety, depression, loneliness, ambivalence, fear, stigma, and concern regarding socioeconomic status (e.g., Gautam et al., 2020; Mukhtar, 2020). Such coping suppression responses were negatively correlated with the sense of well-being and individual, community, and national resilience (Eshel et al., 2020; Kimhi et al., 2021).

Individual resilience constitutes a stable trajectory of healthy functioning after a highly adverse event (Bonanno et al., 2011). Masten (2018) defines it as “the potential of the manifested capacity of a dynamic system to adapt successfully to disturbances that threaten the function, survival, or development of the system,” (P. 187); whereas Seligman and Csikszentmihalyi (2000) regard individual resilience as a process of achieving psychological growth after difficult experiences, and adapting well in the face of adversity. Research has found that individual resilience is positively associated with mindfulness and empathy, and that it is negatively associated with repeated negative thinking (Mathad et al., 2017). Under threats of adversities, such as terror, individual resilience was found to be positively correlated with a sense of coherence and well-being (Eshel and Kimhi, 2016).

Well-being is “an umbrella term for different valuations that people make regarding their lives, events happening to them, their bodies and minds, and circumstances in which they live” (Diener, 2006, p. 400). It is a sense of complete physical, mental, and social well-being and not merely the absence of disease or infirmity (Ryff and Keyes, 1995). One study has concluded that “psychological well-being stands as an important personal resource to favor adaptive coping strategies for academic stress” (Freire et al., 2016).

Well-being is positively associated with individual resilience (Eshel and Kimhi, 2016) and is negatively associated with level of distress (Branson et al., 2019).

Hope is defined as a primarily cognitive, goal-oriented pattern of thought in which people come up with different “pathways” to achieve their goals, remain motivated to follow these pathways, and actively look for alternative pathways to achieve these goals when necessary (Snyder, 2002). Other researchers claimed that hope should be regarded as an experience rather than as an action, since hope is aimed at gaining control over emotions rather than over external circumstances (Herth, 1992). Pleeging et al. (2019) reported moderate to strong correlations of hope measures with overall happiness, life satisfaction, and positive affect measures.

#### Morale

The concept of morale originated in a military context (Sabitova et al., 2020).

According to Weakliem and Frenkel (2006), morale is a general term for positive feelings about prescribed activities of a group. According to Garrett and McNolty (2020) morale is a multifaceted, longitudinal, and relational experience that individuals share when they identify with and contribute to certain kinds of collective activities.

#### Perceived Risks and Observance of Precaution Directives

The required public health preventive behaviors during the COVID-19 pandemic generally involve some sacrifice of personal freedom, and people need to be motivated in order to observe them. Most studies find that health risk perception is significantly correlated with reported adoption of preventative health behaviors such as washing hands, wearing a face mask, and maintaining physical distancing (Dryhurst et al., 2020). We assume that the perceived health and economic risks will positively and significantly predict observing these rules. The perceived importance of health and economic risks in the context of the present plague were compared by international organizations that have found that the probability to get infected with the virus is considered low to moderate by the general population (European Centre for Disease Prevention and Control, 2020), whereas the perceived probability of suffering economic losses is nearly 50% for the global workforce (International Labor Organization, 2020).

The following hypotheses are studied:

1. Contrary to their levels of objective risks, the perceived COVID-19 health risk will be rated by the present student sample as lower than either the perceived security or economic risk, and the perceived security risk will be rated as lower than the perceived economic risk.
2. Levels of perceived health, economic, and security risks will positively and significantly predict the psychological coping suppressing responses of students to anxiety and depression, as well as to perceived academic threats expressed during the COVID-19 pandemic.
3. Levels of perceived health and economic risks will negatively and significantly predict positive psychological responses, such as a sense of well-being, expressed during the COVID-19 pandemic, as well as observance of the required pandemic precaution rules.
4. The three investigated perceived risks will positively and significantly correlate with each other.

## Procedure

Data collection for the student sample took place during the third lockdown in Israel (January 2021) and continued for a period of 2 weeks. A link to the research questionnaire that was prepared by means of the Qualtrics platform was distributed through social networks.

The presentation of the questionnaire indicated that it only aimed at students, and that respondents should indicate the type of academic institution, faculty, and department in which they learned. A single un-reusable link was used in order to avoid multiple participations. The instructions were as follows: “The present questionnaire is aimed at examining students’ attitudes and feelings concerning the current COVID-19 pandemic. Please respond to the following items. This anonymous questionnaire will strictly serve research purposes only. In responding to this questionnaire, you confirm your participation in this research. You may stop responding at any point of time without any consequences.” Although this sample has been distributed across the country and included a wide range of departments and faculties of Israeli colleges and universities, it constitutes, in fact, a convenience sample rather than a representative sample of Israeli students. The sample only included students of recognized Israeli academic institutions.

## Participants

The student sample (*N* = 723) was composed of participants of different ages, most of them were between 18 and 26 years of age. It included more females than males, and mainly secular individuals whose families represented a wide range of income levels. About half of them were first year students. The demographic characteristics of this sample are presented in Table 1.

**TABLE 1:** Distribution of the characteristics of the participants (*N* = 723*).

Variable	Group	Number	%	M
				(SD)
Age	18–25	475	66	26.08 (6.73)
	26–30	160	22	
	31–35	32	5	
	36–40	12	2	
	40 +	35	5	
Gender	Men	193	27	
	Women	525	73	
Religiosity	Secular	498	69	1.42 (0.68)
	Traditional	153	21	
	Religious	69	9.6	
	Very religious	3	0.4	
Political attitudes	Very left	46	6	2.92 (0.91)
	Left	168	23	
	Center	327	46	
	Right	154	22	
	Very right	23	3	
School year	First	373	52	
	Second	197	28	
	Third	104	14	
	Forth and above	39	6	
Faculty	Humanities	379	53	
	Sciences	160	22	
	Did not answer	179	25	
Nationality	Jewish	598	83	
	Arab	94	13	
	Other	26	4	
Family income compare to average in Israel	Much below	132	18	3.18 (1.54)
	Below	124	17	
	Average	156	22	
	Above	176	25	
	Much above	52	7	
	Don’t know	78	11	
Economic support from parents	1. not at all	178	25	2.89 (1.49)
	2. a little	155	22	
	3. medium	103	14	
	4. much	131	18	
	5. very much	150	21	
Economic difficulties due to COVID-19	1. not at all	139	19	2.87 (1.36)
	2. a little	168	23	
	3. medium	179	25	
	4. much	104	15	
	5. very much	126	18	
Employment	1. not employed	367	51	2.27 (1.81)
	2. about 1/3 of time	172	24	
	3. about 1/5 of time	84	12	
	4. about 3/4 of time	42	6	
	5. full time	53	7	

*Because of few partial responses, N varies between 713 and 723.

### Measuring Tools

All the questionnaires on which we based this study were used and validated by us in previous studies. The first eight scales constitute the predicted variables and the last three are the predictors.

#### Depressive and Anxiety Symptoms

Two subscales of the Brief Symptom Inventory (BSI) scale (Derogatis and Melisaratos, 1983; Derogatis and Savitz, 1999) were employed in this study: depression (five items) and anxiety (three items). Because of ethical reasons, the item regarding suicidal thoughts was removed from the scale. The respondents reported the extent to which they are currently suffering from any of the problems presented on the scale. The response scale ranged from 1 = not at all to 5 = to a very large extent, and the internal reliability of this study was high: depression α = 0.86 and anxiety α = 0.71. Adawi et al. (2019) have significantly validated these scales against the level of nomophobia in a sample of healthy Italian volunteers.

#### Perceived Academic Threats

Previous studies have employed detailed questionnaires delineating the academic threats felt by college students because of the COVID-19 pandemic (e.g., Aristovnik et al., 2020; Browning et al., 2021). We asked the respondents to respond to the following single item: “How much do you feel threatened by the academic demands imposed on you while studying under the COVID-19 pandemic conditions**?**” Responses were rated by a five-point Likert scale in which 1 = very low threat and 5 = very high threat. Previous studies have supported the validity of assessing a distinct threat by a single item (e.g., Levkovich and Shinan-Altman, 2020; Kimhi et al., 2021).

#### Individual Resilience

Individual resilience was measured by the 10-item Connor-Davidson (CD-RISC 10, Campbell-Sills and Stein, 2007) scale portraying individual feelings of ability and power in the face of difficulties (Alarcón et al., 2020). Examples of questions are as follows: “I am able to adapt when changes occur”; “I am not easily discouraged by failures.” This scale was rated on a five-point Likert scale ranging from 1 = not true at all to 5 = generally true. The Cronbach’s alpha reliability of this scale in this study was high, α = 0.88. A recent validation showed that, as expected, CD-RISC-10 was positively connected with mental well-being, positive affect, self-esteem, and authentic living, and that it was negatively connected with depressive symptoms, negative affect, acceptance of external influence, and self-alienation (Nartova-Bochaver et al., 2021).

#### Well-Being

The present well-being scale (Kimhi and Eshel, 2009) consisted nine items concerning the perception by individuals of their present lives in various contexts, such as work, family life, health, free time, and others. Responses to these items ranged from 1 = very bad to 6 = very good. This scale has been validated in previous studies. Kimhi et al. (2020a,b) have found in a longitudinal study that level of well-being was positively correlated with individual resilience and hope, and that it was negatively correlated with distress symptoms and sense of danger. The reliability of the scale in this study was found to be high, α = 0.85.

#### Level of Hope

This tool is based on an earlier scale (Jarymowicz and Bar-Tal, 2006; Halperin et al., 2008) that was designed to measure the level of hope for peace among Israel, the Arab nations, and the Palestinians. Its two dimensions are personal and collective hope. The current scale of hope, in the context of the coronavirus crisis, included five items. Two of them refer to the personal level (e.g., “I hope that I will emerge strengthened from the coronavirus crisis”), and three items refer to the collective level (e.g., “I hope that the Israeli society will emerge strengthened from the coronavirus crisis”). The response scale ranged from 1 = very little hope to 5 = high hope. The internal reliability of the scale in this study was high, α = 0.91. A recent study has found that hope, in the context of the COVID-19 pandemic, has been predicted positively by subjective well-being, as well as by individual, community, and national resilience (Kimhi et al., 2021).

#### Morale

The level of personal morale was examined by a single item: “How would you define your morale these days?” The response scale ranged from 1 = not good at all to 5 = very good. Morale level significantly and positively predicted well-being and individual resilience in the COVID-19 pandemic (Eshel et al., 2021).

#### Current Threats

A sense of threat represents the extent to which an individual feels endangered by objects or events from different domains, such as physical, social, psychological, and economic (Kimhi and Eshel, 2009).

The respondents responded to three questions pertaining to the three current potential threat sources: health, security, and economy (e.g., “To what extent do you feel that the current health/security/economic condition personally threatens you?”). The five-point response scale ranged from 1 = not threatening at all to 5 = threatening very much. The path analysis employed for validating the investigated threats in the context of the COVID-19 pandemic showed that both the health and economic threats positively predicted anxiety and depression levels and negatively predicted well-being. Health threat negatively predicted individual resilience as well, and economic threat negatively predicted national resilience (Marciano et al., under review^1^).

#### Compliance With the Pandemic Precaution Rules

Compliance with these precaution rules was assessed by a single question: “To what extent do you comply with the rules aimed at immediately restricting the spread of the pandemic?” Responses to this item ranged from 1 = not at all to 5 = very much.

## Results

The means and standard deviations of the investigated variables are presented in Table 2.

**TABLE 2:** Means and standard deviations of the investigated variables.

Variable (Cronbach’s Alpha)	Mean	Std. Deviation
IR (*a* = 0.88)	3.4716	0.73627
NR (*a* = 0.90)	2.8609	0.84547
Hope (*a* = 0.91)	3.3101	0.96599
Wellbeing (*a* = 0.85)	3.9248	0.87064
danger (*a* = 0.84)	2.8725	0.76979
Anxiety (*a* = 0.71)	2.7610	0.94315
Depression (*a* = 0.86)	2.6142	1.0648
Threat of academic demands	3.19	1.2570

**FIGURE 1:**
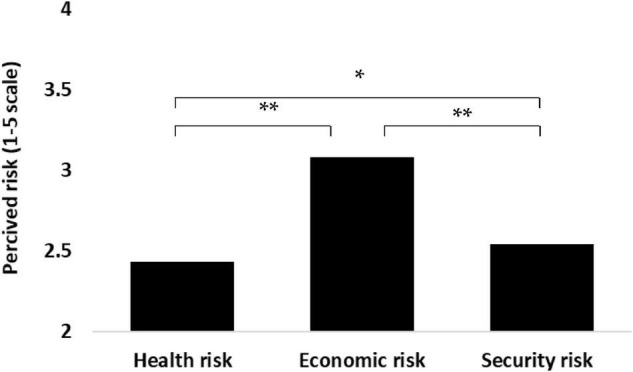
One-way repeated measures analysis of variance (ANOVA) of the three perceived risks. *p < 0.05, **p < 0.0001.

Hypothesis 1 stated that the assessment of the three perceived risks by the investigated student sample will reflect the unrealistic optimism effect, in which the higher the objective risk (i.e., the health risk), the lower will be the rating of the perceived risk. This hypothesis was examined by a repeated measures one-way ANOVA, and was found to be significant [*F*(2, 717) = 108.16, *p* < 0.0001, ηp2 = 0.13]. The results presented in Figure 1 clearly support this hypothesis: The deadliest perceived health risk was rated lowest, next to it was the perceived security risk, whereas the perceived economic risk was rated highest.

Hypotheses 2 and 3 on the predictors of the psychological coping responses by the three threats of health, economy, and security were examined by means of path analysis/Amos Structural Equation Modeling (IBM, SPSS^2^; Arbuckle, 2011). We used maximum likelihood estimates and examined a saturated model, as we did not find any studies that supported an alternative model. It is important to note that in a saturated model, there is no need to examine the fit of the model, as the default and saturated models are the same (Arbuckle and Wothke, 2004). The first saturated model (all paths are examined), which examined hypothesis 2, contained three psychological predictors (perceived health, economic, and security risks) and three coping suppressing indicators: anxiety, depression, and academic threat.

**FIGURE 2:**
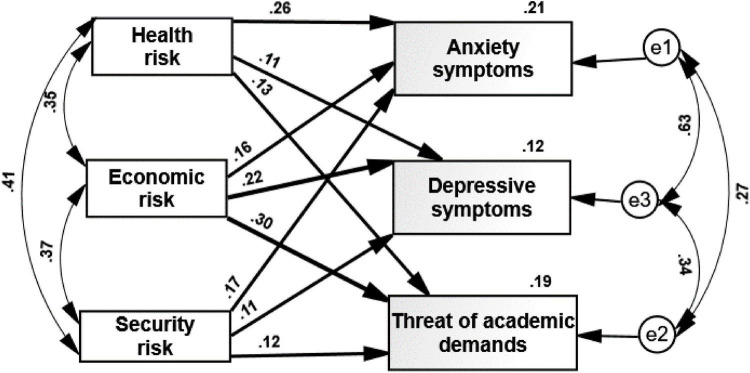
Path analysis for three threats predicting anxiety and depressive symptoms and threat of academic demands. All the paths are significant (*p* < 0.01).

The first path analysis indicated the following (see Figure 2): (a) all paths were significant and positive (*p* < 0.008–0.001); higher threats were associated with higher levels of distress responses. The three predictors explained 21% of the anxiety variance, 12% of the depression variance, and 19% of the academic threat variance. (b) The strongest predictor of level of anxiety was the health risk, whereas the depressive symptoms and the academic threat were more strongly predicted by the perceived economic risk. Security risk was the weakest prediction of these distress reactions. These results fully supported our second hypothesis, and they show further that according to the unrealistic optimism process, the most dangerous health threat is not the best predictor of all the three investigated responses.

The second path analysis, which examined hypothesis 3, included the same three predictors and five predicted responses: well-being, individual resilience, hope, morale, and observance of the caution rules. The results indicated the following (see Table 3): (a) in partial agreement with hypothesis 3, perceived health risk negatively and significantly predicted individual resilience and observance of the caution directives. (b) In full agreement with this hypothesis, perceived economic risk negatively and significantly predicted all the five coping responses. Higher perceived economic risk was associated with lower levels of positive coping responses. (c) In further agreement with hypothesis 3, perceived security risk, significantly and positively predicted well-being but negatively predicted hope. Security risk did not predict significantly individual resilience, morale, and observance of the rules. The three predictors explained 3–9% of these variables. The path analyses also supported hypothesis 4, indicating that the three investigated perceived risks correlated positively and significantly with each other.

**TABLE 3:** Standardized estimates of path analyses for three perceived risks predicting five beneficial reactions.

	Well- being	Individual resilience	Hope	Morale	Observing rules
Health risk	0.09*	−0.16***	−0.08*	−0.08*	0.12**
Economic risk	−0.16***	−0.15***	−0.16***	−0.22***	−0.14***
Security risk	0.11**	−0.06	−0.09*	−0.02	0.03
Explained variance (R^2^)	0.03	0.09	0.07	0.07	0.02

*p < 0.05, **p < 0.01, ***p < 0.001.

As indicated above, the investigated students tended to play down the health danger of COVID-19. A regression analysis examined the extent to which this denial of threat reflected specific demographic characteristics of the participants. The results showed that five of these characteristics (age, religiosity, employment of student, family income, and parental help) had no significant effect on the level of this perceived danger. Gender was the only feature that predicted this tendency: females regarded this threat as more serious than males (*B* = 0.355, SE = 0.094, Beta = 0.14, *t* = 30.765, Sig. = 0). These results replicate in a way the finding of Dolinski et al. (2020) that unrealistic optimism is observed more readily in men than in women.

## Discussion

Unrealistic optimism constitutes a mental process, according to which the level of risk of a major adversity is perceived as less dangerous than the risk of a lesser adversity. According to Shepperd et al. (2013), unrealistic optimism refers to unrealistically positive expectations when compared to an objective criterion, such as an actuarial risk assessment or actual outcomes (e.g., the actual immediate risks of the COVID-19 pandemic compared with the potential future economic and security risks). This study demonstrated a clear case of unrealistic optimism in ratings of the perceived lethal risk of COVID-19 (currently termed as “health risk”). This health risk was perceived as lower than the perceived security risk, and both these risks were rated below the non-lethal perceived economic risk. It has been argued previously that high level of education may protect students from a rather irrational fear of being infected or dying while permitting them to realize the devastating economic, political, and institutional scenarios resulting from the pandemic (Gerhold, 2020). The present respondents were not asked to compare the three perceived risks, and most probably were unaware of the relative ratings assigned by them to these risks. However, in fact, they followed the unrealistic optimism rule that in coping with a highly dangerous threat one should inversely rate the objective risks and assign the lowest rating to the highest risk.

This response is defined as unrealistic optimism. This mode of optimism differs from dispositional optimism, which constitutes a personality trait, or a generalized tendency to expect positive outcomes (Carver et al., 2010). It has been claimed that unrealistic optimism represents a systematic tendency to form beliefs that are biased and often false but have significant benefits, because they increase well-being, contribute to mental and physical health, and support productivity (e.g., Taylor and Brown, 1994; Bortolotti and Antrobus, 2015). Furthermore, Hughes and Zaki (2015) presented evidence that unrealistic optimism is a form of motivated cognition. People process information that is available to them in a way that favors a certain kind of subjectively desirable conclusion. Regarding positive illusions as patterns of beliefs is largely shared in the psychological literature and compatible with common assumptions on how positive illusions work (e.g., Makridakis and Moleskis, 2015; Collard et al., 2016).

We suggest that this process involves partial denial, which was regarded by several researchers (Kirscht et al., 1966; Lazarus, 1983; Horowitz, 1986) as contributing positively to psychological adjustment. Partial denial is very common among patients with physical chronic diseases who are sometimes reluctant to observe health requirements despite being aware of their condition (Livneh, 2009, II). Breznitz (1983) argued that such partial denial may successfully reduce anxiety, stress, and other psychological symptoms, and play a part in raising life satisfaction and adjustment of most people who fear a serious illness. In the present case, the investigated students could have gained psychologically from a less-than-rational response.

The idea that unrealistic optimism perceptions included realistic elements and did not represent the understanding by the respondents of the actual risk of this pandemic was expressed by the retained impact of health risk on some of the investigated psychological responses in this study, which was conducted during the third and most lethal lockdown in Israel due to the COVID-19 pandemic. This impact was not totally impaired despite the low rating, which was assigned to this risk, compared to the economic and security risks.

It is important to note that the perceived health risk was the strongest predictor of level of anxiety but not of depression and academic threats responses, which were best predicted by the perceived economic risk. By the same token, perceived health risk was the best negative predictor of individual resilience, whereas perceived economic risk predicted well-being, hope, and morale better. The high ratings of the perceived economic risk, and its major role in predicting the investigated coping responses could have indicated the poor economic condition of the Israeli students.

A further examination of the present data seems to disagree with this explanation. A large percentage (56.7%) of the investigated students indeed claimed that they were concerned about potential economic difficulties. However, 53.4% of them came from well-to-do families, 53.4% were actually helped economically by their families, and 24.9% of them were employed for half a position or more. It appears that the perceived economic risk indeed reflected unrealistic optimism.

Furthermore, indicators of psychological distress are expected to increase in the face of additional external stressors (Grupe and Nitschke, 2013). Therefore, as expected, perceived anxiety, depression, and academic threats were found to be significantly and positively predicted by all the three perceived threats (health, security, and economic risks). However, only threats that were regarded as actual and realistic risks, that is, health and economic risks, significantly and negatively predicted the positive psychological responses. Well-being and its derivatives, hope and morale, were not significantly predicted by perceived health risk. The COVID-19 vaccination has begun around the time this study was conducted. It is possible that this vaccination campaign has not proven itself yet, and did not reduce the level of the perceived health risk.

In line with hypothesis 4, greater observance of the COVID-19 precaution directives was indeed positively predicted by the level of the perceived health risk. Consistent with the findings of Dolinski et al. (2020), those who regarded health risk as higher more readily adhered to these directives. However, contrary to this hypothesis, perceived economic risk negatively predicted the observance of the rules. As conjectured by Rosman et al. (2020), our study revealed that individuals who fear economic losses to a greater extent were less stringent in observing the closure instructions of staying at home, refraining from work, and maintaining social distancing. This finding may makes sense thinking about the inherent conflicting interests between the precaution rules limitation and making a living, especially for young students.

The COVID-19 pandemic constitutes a collective stressor effect involving health danger with uncertainty, misinformation, and social isolation, which are likely to affect well-being, cause stress, and result in mental disorders (Le et al., 2020; Tran et al., 2020). This pandemic has negatively impacted mental health (Holingue et al., 2020; Wang C. et al., 2020) and caused posttraumatic stress symptoms (Liu et al., 2020). Healthline Mental Health Index (Healthline Media, 2021) indicates further indicates further that up to 45% of adults in the United States have elevated levels of depression and anxiety throughout the COVID-19 pandemic (Rettew et al., 2021). Research on the psychological and behavioral effects triggered by the COVID pandemic has found that personality traits are related to the mental health of an individual in association with this plague. High levels of agreeableness and conscientiousness showed particularly strong associations with better mood during the COVID epidemic period, while higher levels of neuroticism were prominently related to higher levels of perceived stress (Rettew et al., 2021). In particular, in regard to the cascade of psychological and behavioral effects triggered by the COVID pandemic, it has been shown that the negativity of the psychological effects of the lockdown was further modulated by personality traits, alexithymia, and resilience (Osimo et al., 2021). It has also been found that higher alexithymia scores were associated with increased emotional and binge eating as well as higher self-reported physical conditions (Cecchetto et al., 2021).

Research concentrating on coping with major adversities, such as this pandemic, has claimed that the goal of coping research and understanding the relationship between coping processes and long-term adaptational outcomes require an interindividual approach that compares the coping of different individuals with diverse stressful encounters over time (Folkman et al., 1986). Two major theoretical perspectives have analyzed coping styles that are associated with better mental health: the first viewpoint reflects a positive psychology perspective, according to which coping is aimed at developing a reframing strategy, which will re-evaluate a stressful event in positive terms. Coping, according to this position, is aimed at maintaining meaning and purpose in life (Stanislawski, 2019). Research indicates that positive reframing constitutes an effective strategy for reducing both depression (Folkman and Lazarus, 1988; Horwitz et al., 2018) and symptoms of anxiety and stress (Moccia et al., 2020; Wang H. et al., 2020). This coping style seems to reflect the theory of Frankl (1969) that the creation of meaning is crucial for people to transcend tragic circumstances. His influential ideas have earned empirical support, showing that finding meaning has an important role in the psychological recovery from an adversity (Updegraff et al., 2008). A second theoretical position (Folkman et al., 1986) has claimed that emotion-focused coping is any strategy used to reduce stress and tension by regulating the state of emotion. This definition agrees with the claim of stress theories that the major function of coping is to reduce fear and anxiety, raised by a stressful condition, and restore adjustment (Breznitz, 1983; Lazarus, 1983). Research has shown that individuals who have used more often emotion-focused coping in the first phase of the COVID-19 pandemic were likely to experience less anger and sadness throughout this pandemic (Malesza, 2021).

Unrealistic optimism, which constitutes a form of emotion-focused coping, is aimed at reducing the fears raised by the COVID-19 pandemic by changing its perceived risk. This coping mode follows the analysis of Breznitz, which indicates that anxiety and fear may be reduced by seven aspects of partial denial: denial of information, denial of threatening information, denial of personal relevance, denial of urgency, denial of vulnerability/responsibility, denial of affect, and denial of affect relevance (Breznitz, 1983). This partial denial is used in a process of restructuring perceived threats. The prevalence of unrealistic optimism (Shepperd et al., 2013) seems to support the claim that rather than being a pathological response, it is an emotion-focused process aimed at supporting individual adjustment to harmful and traumatic external events, and may be considered as an adaptive behavior, supporting the resilience of people (Horowitz, 1986; Alloy, 2017). This study indicates its meaningful role in coping with the COVID pandemic. A major claim could have been that age influenced the three perceived investigated threats, and especially the health threat, which supposedly endangers older individuals to a greater extent. An examination of this issue shows no significant correlation between age of the participants and their perceived health, economic, and security threats. A further examination of the present data indicated that, in agreement with previous findings (European Centre for Disease Prevention and Control, 2020; International Labor Organization, 2020), economic threat should not be regarded as a secondary danger in the context of the COVID-19 pandemic compared to health risk. On the contrary, our data show that economic threat (mean = 3.08, SD = 1.221) was regarded by the present sample as higher than the health threat (mean = 2.43, SD = 1.118). The difference between these means is highly significant (*t* = 12.999).

### Limitations

The main limitation of this research and other studies based on questionnaires is the lack of objective measures in terms of health, economic, and safety risks, since these studies are based on perceptions of the respondents. The second limitation pertains to sampling by means of an internet sample. Even though the sample is large and includes a wide variety of demographic variables and a wide range of Israeli academic institutions, there is no guarantee that it is a representative sample of Israeli students.

## Conclusion

Several conclusions can be derived from the present findings. First, unrealistic optimism should be expected in perceived assessments of the health risk of COVID-19 pandemic, as well as in coping with other related risk conditions. This unrealistic optimism represents perceptions of college students as well as those of the general public, as indeed was found by a large number of studies (e.g., Lanciano et al., 2020). Second, psychological coping responses expressed during the COVID-19 pandemic did not represent only the health threat of the pandemic, as was hypothesized by previous research (e.g., Krok and Zarzycka, 2020; Lanciano et al., 2020). They reflected concurrently other threats sensed by the general public. Third, these perceived threats predicted differentially varied psychological coping responses: perceived health risk was not always the stronger predictor of all the psychological responses, and perceived security risk predicted negative responses but not beneficial responses. Fourth, unrealistic optimism was often regarded as an irrational way of assessing threats, while the present data show that this type of optimism also includes logical and realistic elements. The inverse ratings of the perceived risk levels compared to the objective risk levels were not necessarily an unreasonable judgment. It is quite possible that this inversion was motivated by a kind of adaptive partial denial of risks that provided a valuable contribution to reducing anxieties and supporting the resilience of individuals in the face of an extensive threat. Fifth, college students, much the same as the general public, encountered a substantial number of stressful events because of the COVID-19 pandemic, which is still raging in different countries. These students must cope with a host of difficulties and face many concerns pertaining to their ability to complete their studies and achieve an academic degree. Further studies are required in order to examine two additional research directions: (a) Identifying additional risks that may increase the anxiety of students, as well as motivational elements that will enhance their sense of well-being and increase their coping abilities. (b) While the responses of the investigated student were not significantly affected by most of their demographic characteristics, it should be examined whether such attributes influence the responses of other age groups in different cultural settings. In more general terms, understanding the mechanisms underlying the impact of the COVID-19 on mental health is essential to developed novel interventions to protect mental well-being from stressful conditions involved, to diminish a potential mental health epidemic associated with the current COVID-19 pandemic, and to promote more effective coping styles across populations.

Further research is recommended to substantiate the role of optimistic bias in explaining the reduced perceived jeopardy of the COVID-19 pandemic. Bottemanne et al. (2020) have delineated several alternative explanations that have been offered to decrease the perceived danger of this pandemic, which do not include unrealistic optimism. Research shows that people tend to underweight the probable consequences of the pandemic when adopting precautionary behaviors (Barron and Yechiam, 2009). Furthermore, people vary on how much they discount risks, whenever they perceive them as still temporary or distant (Peake, 2017). Putting in place strict mandatory measures of social distancing may involve psychological, social, and economic costs. Avoiding these short-term risks could come at the expense of long-term health benefits of containing the pandemic (Thaler et al., 1997).

## Data Availability Statement

The raw data supporting the conclusions of this article will be made available by the authors, without undue reservation.

## Ethics Statement

The studies involving human participants were reviewed and approved by Ethics Committee, Tel Aviv University. The patients/participants provided their written informed consent to participate in this study.

## Author Contributions

SK and BA: conceptualization and supervision. SK and YE: methodology, formal analysis, and data curation. BA and HM: validation. YE, SK, HM, and BA: investigation and resources. YE: preparation and writing of original draft and visualization. SK, HM, and BA: writing review and editing. SK: project administration. All authors contributed to the article and approved the submitted version.

## Conflict of Interest

The authors declare that the research was conducted in the absence of any commercial or financial relationships that could be construed as a potential conflict of interest.

## Publisher’s Note

All claims expressed in this article are solely those of the authors and do not necessarily represent those of their affiliated organizations, or those of the publisher, the editors and the reviewers. Any product that may be evaluated in this article, or claim that may be made by its manufacturer, is not guaranteed or endorsed by the publisher.

## Footnotes

1 Marciano, H., Eshel, Y., Kimhi, S., and Adini, B. (2021). Hope and fear of threats as predictors of coping with two major adversities, the COVID-19 pandemic and an armed conflict (under review).

2
https://www.ibm.com/il-en/marketplace/structural-equation-modeling-sem
